# Correlation Between Personal Health History and Depression Self-Care Practices and Depression Screening Among African Americans With Chronic Conditions

**DOI:** 10.5888/pcd15.170581

**Published:** 2018-12-06

**Authors:** Priscilla A. Barnes, Tilicia L. Mayo-Gamble, Doshia Harris, David Townsend

**Affiliations:** 1 Indiana University School of Public Health-Bloomington, Bloomington, Indiana; 2 Georgia Southern University, Statesboro, Georgia; 3 Af-Am Consulting, Indianapolis, Indiana

## Abstract

Little is known about the influence of personal health history and depression self-care practices on screening for depression by health care providers among African Americans with chronic conditions. African Americans (N = 203) aged 18 years or older and living with at least one chronic health condition in a metropolitan city completed a 45-item community perceptions survey. The number of depression symptoms experienced per month was positively associated with screening for depression by a health care provider; perceived ability to identify depression symptoms was inversely associated with screening by a health care provider. Understanding patients’ health history and self-care practices can initiate provision of information or support services to improve patient–provider communication about depression.

## Objective

Forty-eight percent of adults in the United States living with one or more chronic conditions (eg, heart disease, cancer, diabetes, mood disorders) are African American (1,2). An area of concern is the co-existence of depression with a physical condition (3), but symptoms of depression often go unrecognized (4,5). Moreover, African Americans may be reluctant to discuss symptoms with health care providers for fear of being stigmatized (5–8). 

We examined whether personal health history and depression self-care practices were associated with depression screening by health care providers among African Americans with chronic conditions. An analysis of community perceptions can inform development of culturally tailored messages encouraging patient–provider dialogue during medical appointments.

## Methods

A convenience sample of 203 African Americans completed a cross-sectional survey about mental health services that was administered from January through April 2014. Inclusion criteria were being aged 18 years or older, having one or more physical chronic conditions, and living in Indianapolis at the time of survey distribution. Institutional review board approval (protocol no. 1312966930) was granted from Indiana University.

The survey consisted of 45 questions that measured indicators related to physical and mental health. Individuals’ personal experiences accessing mental health services were also assessed. A panel of experts working in a primary care office, at a hospital mental health department, and at the state National Black Nurses Association reviewed the survey before distribution. Eligible participants at community centers, places of worship, barber shops, and community events completed the survey in approximately 10 minutes and received an incentive.

Of the 45 questions, 18 focused on depression screening, personal health history, and depression self-care practices. The outcome variable was having ever been screened for depression by a health care provider. Personal history with depression included number of poor mental health days and number of depression symptoms per month. Perceptions toward depression self-care were assessed by 1) being able to make an appointment, 2) knowing who to call for personal or emotional problems, 3) being able to identify symptoms of depression, 4) being able to take an antidepressant medicine, 5) being able to make oneself feel better, and 6) being able to avoid difficult situations that can trigger depression. Perceptions were measured on a 5-point Likert scale of agreement (from 1 being strongly disagree to 5 being strongly agree). Participants provided demographic information, including sex, annual household income, education level, employment status, marital status, age, general health status, health insurance status, and number of chronic health conditions.

We calculated descriptive statistics on all variables. Pearson correlation analysis determined which demographic characteristics to include in the logistic regression analysis. Binary adjusted logistic regression determined factors associated with depression screening. Data were analyzed using SPSS version 23 (IBM Corporation). Significance was set at *P* < .05.

## Results

Complete questionnaires were returned by 138 (68%) women and 65 (32%) men (Table 1). Approximately 37% (n = 75) of participants earned less than $10,000 per year, 58% (n = 118) had a high school diploma/general educational development certificate or some college, and 87% (n = 176) were insured. The mean age of participants was 53.9 years. Fifty eight percent (n = 118) reported never having been screened for depression. On average, participants had 2 chronic conditions and 2 symptoms of depression per month.

**Table 1:** Demographic and Health Outcome Data of Study Population (N = 203)^a^, Study on Correlation Between Personal Health and Depression Self-Care Practices and Being Screened for Depression Among African Americans, Community Perceptions Survey, Indianapolis, Indiana, 2014

Characteristic/Outcome	Value
**Sex**	
Female	138 (68.0)
Male	65 (32.0)
**Mean age, y (SD)**	53.9 (14.79)
**Income, $**	
<10,000	75 (36.9)
10,000–19,999	41 (20.2)
20,000–29,999	24 (11.8)
30,000–39,999	21 (10.3)
40,000–49,999	15 (7.4)
≥50,000	16 (7.9)
**Education**	
Less than high school	44 (21.7)
High school diploma/general educational development certificate	57 (28.1)
Some college	61 (30.0)
Technical school/college graduate	35 (17.2)
**Employment**	
Does not work	78 (38.4)
Employed	76 (37.4)
Student	8 (3.9)
Retired	41 (20.2)
**Marital status**	
Married	40 (19.7)
Divorced or separated	67 (33.0)
Widowed	24 (11.8)
Single	69 (34.0)
**General health status**	
Very good	29 (14.3)
Good	80 (39.4)
Fair	77 (37.9)
Poor	17 (8.4)
**Health insurance status**	
Insured	176 (86.7)
Not insured	27 (13.3)
**Screened for depression**	
Yes	82 (40.4)
No	118 (58.1)
**Health outcome, mean (SD)**	
No. of chronic conditions	2.05 (1.32)
No. of times visited a doctor per month	5.07 (7.07)
No. poor mental health days per month	5.91 (8.94)
No. of depressive symptoms per month	2.04 (1.89)

Abbreviation: SD, standard deviation.

a Values are no. (%) unless otherwise indicated.

Demographic characteristics (income, employment, and number of chronic conditions) were not statistically associated with depression screening. Income and employment were negatively correlated with depression screening (*r *= −0.15, *P* = .04 and *r* = −0.24, *P* = .001). Participants who reported having one or more chronic conditions or self-identified symptoms of depression were more likely to be screened by a health care provider (Table 2). Number of chronic conditions was positively correlated with depression screening (*r* = .31, *P* < .001). For personal history with depression, results indicated that for one unit increase in the number of depression symptoms per month, participants were more likely to be screened for depression (odds ratio [OR] [95% confidence interval (CI)] = 1.71 [1.10–2.66]). Number of mental health days per month was not associated with depression screening. Among perceptions toward depression self-care measures, ability to identify symptoms of depression was associated with depression screening. For each increase on the perceived ability to identify symptoms of depression (ie, ability to identify symptoms of depression) participants were less likely to be screened for depression (OR [95% CI] = 0.27 [0.89–4.83]). 

**Table 2:** Logistic Regression of Socio-Demographic Factors Associated with Depression Screening, Study on Correlation Between Personal Health and Depression Self-Care Practices and Being Screened for Depression, Community Perceptions Survey, Indianapolis, Indiana, 2014

Variable	Odds Ratio (95% Confidence Interval)	Standard Error	*P* Value
**Income, $**			
<10,000	1 [Reference]		
10,000–19,999	0.78 (0.13–4.64)	0.71	.79
20,000–29,999	0.74 (0.07–8.06)	0.90	.81
30,000–39,999	1.55 (0.12–2.90)	2.06	.74
40,000–49,9999	0.44 (0.04–4.61)	0.52	.49
≥50,000	2.19 (0.21–2.04)	2.63	.51
**Employment**			
Unemployed	1 [Reference]		
Employed	0.38 (0.08–1.98)	0.32	.25
Student	0.60 (0.03–1.92)	0.89	.73
Retired	0.76 (0.09–6.75)	0.85	.81
**Number of chronic conditions**	1.30 (0.76–2.23)	0.36	.33
**Personal history with depression**			
Number of mental health days	0.999 (0.91–1.09)	0.04	.99
Number of symptoms per month	1.71 (1.10–2.66)	0.38	.02
**Perceptions toward depression self-care**			
How to make an appointment . . . get help	1.19 (0.35–1.98)	0.58	.72
Know who to call to get help right away	0.83 (0.07–0.99)	0.37	.68
Can you identify symptoms of depression	0.27 (0.89–4.83)	0.18	.049
How to take antidepressant medication or get counseling	2.08 (0.52–4.58)	0.89	.09
Make myself feel better by doing more pleasurable activities	1.55 (0.51–3.08)	0.86	.43
Can avoid difficult situations that can trigger depression	1.26 (0.05–2.58)	0.58	.62

## Discussion

This formative research offers new perspectives to explore help-seeking behaviors among African Americans with pre-existing chronic conditions. Mental health days per month is a vague concept that may be perceived as involving extreme fatigue (eg, “I need a mental health day.”). Conversely, number of depression symptoms focuses on a specific condition and may prompt the patient or provider to inquire whether chronic condition(s) or depression are affecting daily activities. More research should be conducted on the meaning of these concepts from the perspective of the African American community. Culturally relevant messages can be developed to promote “check-ins” that prompt discussion as opposed to reprimand for noncompliant behavior.

Participants in this study, on average, visited a medical provider 5 times per month, which may place this sample at a higher probability of being screened. This factor is important given that depression screening is dependent on seeing a health care provider. Despite this finding, increased confidence to self-identify symptoms of depression equated to decreased likelihood that participants would talk to their medical provider. Studies demonstrate that African American patients do not initiate discussion because of perceptions that disclosure within primary care is not appropriate, fear of not having a choice in treatment decisions, and the emotional cost of talking about symptoms (9–11). Clinical–community partnerships involving African American churches can focus on creating culturally relevant spaces to conduct depression screenings.

Our study has limitations. First, the sample size was small, so findings cannot be generalized to the broader community. Second, we used self-reported data, which may be inaccurate because of recall bias or respondent bias. Third, data are were cross sectional, so causality could not be inferred. These limitations, however, do not outweigh the contribution of the study. This exploratory study underscores the necessity of exploring sociological factors that affect the initiation of preventive screenings in health care settings.

## References

[1] Centers for Disease Control and Prevention. African American Health. https://www.cdc.gov/vitalsigns/aahealth/index.html. Accessed July 29, 2017.

[2] Trust for America’s Health. Improving the health of low-income and minority communities. http://healthyamericans.org/assets/files/10ThingsMinorities.pdf. Accessed November 4, 2017.

[3] Chapman DP , Perry GS , Strine TW . The vital link between chronic disease and depressive disorders. Prev Chronic Dis 2005;2(1):A14. 15670467 PMC1323317

[4] Bazargan M , Hamm-Baugh VP . The relationship between chronic illness and depression in a community of urban black elderly persons. J Gerontol B Psychol Sci Soc Sci 1995;50(2):S119–27. 10.1093/geronb/50B.2.S119 7757840 PMC4909475

[5] Dunlop DD , Song J , Lyons JS , Manheim LM , Chang RW . Racial/ethnic differences in rates of depression among preretirement adults. Am J Public Health 2003;93(11):1945–52. 10.2105/AJPH.93.11.1945 14600071 PMC1199525

[6] Gitlin LN , Chernett NL , Dennis MP , Hauck WW . Identification of and beliefs about depressive symptoms and preferred treatment approaches among community-living older African Americans. Am J Geriatr Psychiatry 2012;20(11):973–84. 10.1097/JGP.0b013e31825463ce 22643600 PMC4030409

[7] Green KM , Fothergill KE , Robertson JA , Zebrak KA , Banda DR , Ensminger ME . Early life predictors of adult depression in a community cohort of urban African Americans. J Urban Health 2013;90(1):101–15. 10.1007/s11524-012-9707-5 22689296 PMC3579304

[8] Sellers SL , Bonham V , Neighbors HW , Amell JW . Effects of racial discrimination and health behaviors on mental and physical health of middle-class African American men. Health Educ Behav 2009;36(1):31–44. 10.1177/1090198106293526 17130248

[9] Williams DR , Yan Yu , Jackson JS , Anderson NB . Racial differences in physical and mental health socio-economic status, stress and discrimination. J Health Psychol 1997;2(3):335–51. 10.1177/135910539700200305 22013026

[10] Keller AO , Valdez CR , Schwei RJ , Jacobs EA . Disclosure of depression symptoms in primary care: a qualitative study of women’s perceptions. Womens Health Issues 2016;26(5):529–36. 10.1016/j.whi.2016.07.002 27531601 PMC5026909

[11] Bailey RK , Patel M , Barker NC , Ali S , Jabeen S . Major depressive disorder in the African American population. J Natl Med Assoc 2011;103(7):548–59. 10.1016/S0027-9684(15)30380-1 21999029

